# Original Adverse Outcome Pathway linking neuronal exposure to nanoparticles to the onset of Alzheimer’s disease

**DOI:** 10.1002/alz.087445

**Published:** 2025-01-03

**Authors:** Benoit Schneider

**Affiliations:** ^1^ Ecole polytechnique ‐ CNRS UMR7654, Palaiseau, Ile‐de‐France, France; Université Paris Cité ‐ Inserm UMR‐S1124, Paris, Ile‐de‐France France

## Abstract

Alzheimer’s disease (AD) is the most common dementia in humans that today concerns 50 million individuals worldwide and will affect more than 100 million people in 2050. Except for familial AD cases (<5% of AD patients) for which AD pathology connects to mutations in critical genes involved in the processing of the amyloid precursor protein into neurotoxic Aß peptides, it remains unknown what provokes the overproduction and deposition of Aß peptides in the brain of sporadic AD cases (>95% of AD patients). Some nanosized materials, e.g., nanoparticles (NPs), are suspected of playing a role in the growing incidence of AD, due to their reactivity with biological systems, easiness of crossing physiological barriers, capacity to reach the central nervous system and accumulate in the brain. Incriminated NPs are the ultrafine air‐borne particulate matter and manufactured NPs, such as titanium dioxide (TiO_2_) NPs widely used in food and cosmetic industries or carbon black (CB) NPs used in rubber and as black pigment. Both TiO_2_‐ and CB‐NPs display neurotoxicity, but the mechanisms by which those NPs affect neuronal homeostasis and place neurons on the path to degeneration are unknown.

Combining *in vitro* and *in vivo* approaches, we provide prime evidence that TiO_2_‐ and CB‐NPs bind a plasma membrane neuronal receptor, the cellular prion protein PrP^C^, well‐known for its implication in prion diseases, and corrupt PrP^C^ signaling activity. Dysregulation of PrP signaling by TiO_2_ ‐ or CB‐NPs pomotes the NADPH oxidase‐mediated overproduction of reactive oxygen species that alter neuronal redox equilibrium and the activation of 3‐phosphoinositide‐dependent kinase 1, at the root of an increased vulnerability of NP‐exposed neurons to inflammation and the overproduction of neurotoxic Aß peptides. By showing that neuronal exposure to some TiO_2_‐ and CB‐NPs induce molecular signs of AD, these mechanistic data provide new insight into how human exposure to some engineered and environmental NPs may predispose to neurodegenerative diseases.

Identifying the mechanisms by which nanoparticles and other pollutants alter neuronal homeostasis would enable the generation of a database of pollutant‐associated Adverse Outcome Pathways and provide clues for counteracting the adverse effects of those pollutants on the central nervous system.

